# Persistent Post-Traumatic Headache and Migraine: Pre-Clinical Comparisons

**DOI:** 10.3390/ijerph17072585

**Published:** 2020-04-09

**Authors:** Matilde Capi, Leda Marina Pomes, Giulia Andolina, Martina Curto, Paolo Martelletti, Luana Lionetto

**Affiliations:** 1 Department of Neurosciences, Mental Health & Sensory Organs (NESMOS), Sapienza University of Rome, 00185 Rome, Italy; matildecapi@gmail.com (M.C.); ledama@hotmail.it (L.M.P.); 2 Department of Biomedical and Dental Sciences and Morphofunctional Imaging, University of Messina, 98122 Messina, Italy; giulia.andolina90@virgilio.it; 3 Department of Human Neurosciences, Sapienza University of Rome, 00185 Rome, Italy; martina.curto@gmail.com; 4 International Mood & Psychotic Disorders Research Consortium, Mailman Research Center, McLean Hospital, Belmont, MA 02478, USA; 5 Department of Mental Health, ASL Roma 3, 00125 Rome, Italy; 6 Sant’Andrea Hospital, Regional Referral Headache Centre, 00189 Rome, Italy; 7 Department of Clinical and Molecular Medicine, Sapienza University, 00189 Rome, Italy; 8 Mass Spectrometry Lab-Clinical Biochemistry Unit, Sant’Andrea University Hospital, 00189 Rome, Italy; luanalionetto@gmail.com

**Keywords:** PTTH, migraine, persistent post-traumatic headache, pathophysiology, drugs, traumatic-brain injury

## Abstract

*Background:* Oftentimes, persistent post traumatic headache (PPTH) and migraine are phenotypically similar and the only clinical feature that differentiate them is the presence of a mild or moderate traumatic brain injury (mTBI). The aim of this study is to describe the differences in brain area and in biochemical cascade after concussion and to define the efficacy and safety of treatments in use. *Methods:* Sources were chosen in according to the International Classification of Headache Disorder (ICHD) criteria. *Results:* The articles demonstrated a significant difference between PPTH and migraine regarding static functional connectivity (sFC) and dynamic functional connectivity (dFC) in brain structure that could be used for exploring the pathophysiological mechanisms in PPTH. Many studies described a cascade of neuro-metabolic changes that occur after traumatic brain injury. These variations are associated to the mechanism occurring when developing a PPTH. *Conclusions:* The state of art of this important topic show how although the mechanisms underlying the development of the two different diseases are different, the treatment of common migraine is efficacious in patients that have developed a post traumatic form.

## 1. Introduction

The International Classification of Headache disorder second edition (ICHD-2) [1] classifies migraine as a primary headache disorder and post-traumatic headache (PTH) as secondary disorder following a trauma or injury [2,3]. In particular, accordingly to the ICHD-2, the PTH has to onset within seven days of traumatic injury to the head and/or neck or within 7 days of recovering consciousness and the ability to report headache [4]. The third edition (ICHD-3) provides an implementation of post traumatic headache adding the definition of persistent post-traumatic headache (PPTH). Considerably, ICHD-3 divided PPTH in “Persistent headache attributed to moderate or severe traumatic injury to the head” and “Persistent headache attributed to mild traumatic injury to the head” [5]. PTH is defined as acute if it develops during the first three months from trauma and as persistent if this condition continues for longer than three months. It is important to note how PTH occurs more easily after mild traumatic brain injury (mTBI) rather than moderate or severe TBI [6]. The clinical picture of PTH is more defined in subjects who report headache directly after the trauma and with no history of migraine, even though it is not clear whether the trauma induces PTH or triggers a pre-existing susceptibility to the migraine itself. Genetic predisposition might play a role, even if not clear yet, in the onset of PPTH, considering that genes associated with familial hemiplegic migraine type 1 and 2 induce an increase in neurological responses following trauma [7]. Several studies have found that patients with a family history of headache exhibit a migraine phenotype more likely compared to patients who do not have this familiar history [7,8,9]. The onset time of headache after trauma can be useful for the diagnosis of PTH, considering that PTH, tension-type headache and migraine symptoms are rather similar. ICHD-3 classification defined PTH a disorder characterized by dizziness, fatigue, low ability to focus, motor and sleep disorder, anxiety, irascibility, memory problems and changes in personality.

Patients with PTH present decreased activity levels due to the increase in headache pain, cranial hyperalgesia and frequency of headaches crises [9]. Several factors might be involved in the development of PTH, such as axonal injury, alteration in brain metabolism and/or haemodynamics and genetic stability [10]. Moreover, accordingly to ICHD-3 classification, PPTH facilitates the misuse of acute headache medication and unsuccessful preventative PTH pharmacological treatment could lead to the medication-overuse condition (MO) [11]. Several studies conducted at the Danish Headache Center have demonstrated that patients with PPTH met the criteria for MO, even if in many patients the occurrence of headache did not improve following detoxification therapy [12,13]. In both cases (PPTH and MO), the possibility that the treatments are ineffective and induce chronicity associated with an overuse of medicaments could be sought in a non-optimal choice of the drug. The target of this therapeutic failure to date seems to be the personalized approach [14]. This review points to evaluate similarities and differences between migraine and PPTH, mainly considering the pathophysiology and the pharmacological treatment.

## 2. Methods

The protocol for this systematic review has been designed following scoping searches of the available literature. We also reviewed the reference lists of relevant primary articles. The review process followed the Preferred Reporting Items for Systematic Reviews and Meta-Analyses (PRISMA) guidelines.

### 2.1. Eligibility Criteria

Researches were considered eligible if they met the main criteria: randomized controlled trials of adult participants diagnosed with post traumatic headache or persistent post traumatic headache based on defined clinical criteria. Case–control studies, cohort studies or observational studies were included as reference for this review. The exclusion criteria included unclearly defined study design, target population or unclear results. The search was limited to articles on human subjects published in English.

### 2.2. Source of Information

We systematically searched PubMed, Clinicaltrial and Researchgate until 2020. In addition, research was carried out in specific scientific journals, finding potentially eligible studies for the writing of this review: *Headache; The Journal of Headache and Pain, Cephalalgia; Brain, Behavior, and Immunity; Clinical Neurology and Neurosurgery; Neurology*, among others.

### 2.3. Search Strategy

The search was performed with the following search string: migraine; migraine therapy; migraine AND persistent post-trauma headache; persistent post-trauma therapy; post traumatic headache OR post-traumatic headache OR posttraumatic headache OR post traumatic migraine.

The electronic database search was supplemented with manual searches for published, unpublished and ongoing randomized clinical trials (RCTs) in ClinicalTrials.gov. The search term was “migraine and persistent post-traumatic headache.”

## 3. Results

Based on the research of the topics carried out, both original articles and reviews have been taken into consideration. The focus is on articles dealing with the sensory differences between migraine and PPTH, the neurophysiopathological and biochemical mechanisms and the pharmacological treatments. The papers deal with experiments conducted both on animal models and people, including children and adolescents.

### 3.1. Sensory Differences between Migraine and PPTH

Although migraine and PPTH present similar symptoms, only a few studies focused attention on structural differences of the brain and on sensory profile that mark migraine and PPTH. Dumkrieger et al. focused their attention on the differences in brain structure of patients suffering from migraine and PPTH through a magnetic resonance imaging analysis. They analyzed the static functional connectivity (sFC), the correlation between two signals in the region of interest (ROI), the dynamic functional connectivity (dFC) or a connectivity/correlation that varies with time [15]. Significant differences in sFC and dFC have been found between migraine and PPTH for the regions that participate in the elaboration of pain, including the somatosensory region and the hypothalamus. The dFC was found to be significantly correlated with the frequency of headache for the PPTH group and the intensity of pain for the migraine group. sFC and dFC may therefore be useful for exploring the pathophysiological mechanisms in PPTH and migraine [16]. Although it is hard to connect preclinical works and patients’ experience, visual stimuli could accentuate the migraine pain following the physiological connection existing between the sensory pain processing region and the visual processing region. Levy et al. have studied the sensory profile that characterized patient suffering of PPTH after TBI. The study was conducted exclusively on male military personnel that have been grouped in three groups: PPTH with migraine-like symptoms, PPTH with tension type headache and a last group with recurring headache (TTH) [17]. The military with PPTH were further divided into two subgroups based on intensity, localization, aggravating and mitigating factors for headaches: PPTH with a phenotype similar to migraine (PPTH-migraine) group and PPTH similar to tension-type headache (PPTH-TTH) group, respectively. Photophobia and stress without photophobia were considered as contributing factors to a worsening of PPTH-migraine and PPTH-TTH (*p* = 0.006 and 0.0005, respectively); quiet conditions and analgesic treatment contributed to significantly improve both PPTH-migraine and PPTH-TTH (*p* = 0.049 and 0.02, respectively). Darkness and high volume were considered respectively attenuating and worsening factors in both PPTH subgroups; similarly, head–neck pain and the undefined localization of pain were reported as common factors in both types of headache [18].

### 3.2. Biochemical Alteration and Pathophysiology

**Figure 1:**
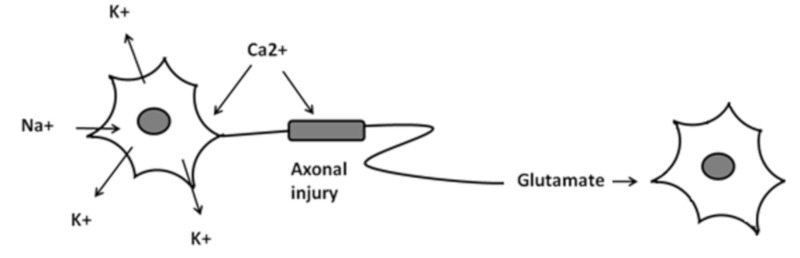
Metabolic alteration in Post Traumatic Headache disorder.

Many studies have shown that the migraine pain pathway was triggered by the activation of the trigemino-vascular system with the consequent release of several inflammatory neuropeptides. TBI leads to both cellular and axonal metabolic alterations; these alterations follow an increase in extracellular potassium concentrations, intracellular sodium and calcium levels, glutamate levels (Figure 1). The restoration of ionic homeostasis requires such quantities of Adenosine Triphosphate (ATP) as to reduce its resources on a cellular level. Consequently, the mismatch of supply and demand of ATP leads to an accumulation of lactate and oxidative stress status [19]. Increases in the concentration of intracellular calcium trigger a cascade that leads to the collapse of the neurofilament with disassembly of the microtubules. This results in axonal damage, known as secondary axotomy, which might be part of the pathobiology of mTBI [20,21]. The neuroinflammation after TBI might induce excitability of the central nervous system. The aura phase of migraine is thought to result from cortical spreading depression (CSD), i.e., transient and self-propagating depolarization in glial cells and neurons [22]. CSD leads to an increase in extracellular potassium concentration and glutamate levels, which follows the activation of the trigeminal system [23,24] that is involved in migraine [25,26]. CSD upregulates matrix metal proteinases (MMPs) that exhibit elevated levels in migraine-induced neuroinflammation or TBI [21,27]. The involvement of neuroinflammation in the pathophysiology of PPTH has been demonstrated in preclinical studies in which N-acetyl cysteine (NAC) showed anti-inflammatory and neuroprotective effects [28,29]. Treatment with NAC in military patients with mTBI led to an 86% reduction in symptoms within seven days from the start of the treatment, compared to placebo [30]. Although the pathophysiological mechanisms underlying migraine and PPTH are not yet fully understood, several data suggest that migraine and its chronic forms as drug-induced migraine showed altered levels of oxidative stress biomarkers and a reduction in antioxidant mechanisms [31]. Migraine patients have compromised mitochondrial metabolism, which affects both neural tissue and the production of peripheral markers of oxidative stress [32]. Oxidative metabolism turns out to be altered in patients with migraine, with or without aura, which also causes higher levels of lactic acid [33] and a deficit of Nicotinamide Adenine Dinucleotide plus Hydrogen (NADH)-dehydrogenase and cytochrome-c-oxidase [34]. As widely known, an abuse of migraine-drugs induces an increase of neural excitability at the cortex and trigeminal system level, thus increasing the perception of pain [35], increasing the abuse of drug and reducing the antioxidant capacity, as evidenced by the reduction of the levels of Ferric Reducing Antioxidant Power (FRAP) plasmatic and total thiol groups. This suggested reduction in antioxidant capacity could be in accordance with the altered energy metabolism in the brain [32]. Hypothetically, PPTH could be associated with both a greater mitochondrial dysfunction and an imbalance between the oxidative and antioxidant status, if compared to migraine [31]. Human-induced migraine studies have determined that Calcitonin Gene Related Peptide (CGRP) upregulate cyclic Adenosine Monophosphate (cAMP) levels, playing a key role in the pathogenesis of both migraine and PPTH [36]. CGRP is a potent vasodilator [37,38], whose receptors are present both in nerve fibers and in trigeminal neurons and in many areas involved in nociception and pain processing [39,40]. The release of CGRP, following trigeminal stimulation, induces neurogenic inflammation with mast cell degranulation [41], the release of pro-inflammatory modulators and the alteration of the blood-brain barrier [42]. The stimulation of the trigeminal system induces an increase in blood levels of CGRP causing attacks of migraine similar to those of spontaneous migraine [43]. Therefore, CGRP-dependent mechanisms are involved in central awareness in both migraine and PPTH [44]. An increase in blood CGRP was also noted in a study of rodents suffering from drug abuse migraines [45,46], linked to an increase in extracellular and cephalic allodynia (CA). In man, cephalic allodynia has been found mainly in patients with PPTH [47], although similar results have also been found with migraine [48]. Activation and sensitization of the trigeminal caudal nucleus (TCN) were considered as mechanisms that underlie allodynia, and it is possible that the sensitization of thalamic neurons is at the basis of extracellular allodynia [49].

## 4. Treatment Approach

The approach to the treatment of PTH may vary considerably among clinicians. Non-pharmacological approaches in patients with PTH with migraine-like features involve lifestyle modifications, such as exercise, good sleep, hydration and management of stress or events triggering migraine attacks. The pharmacological treatment for PTH uses acute or preventive medications typical of primary headache disorders. The Food and Drug Administration did not approve treatments for PTH with migraine phenotype with a high probable of unnecessary therapy, and the pharmacological treatment may interfere with comorbidities such as depression and anxiety.

Regarding comorbidities, anxiety and depression were diagnosed in soldiers with TBI or post-traumatic stress disorder (PTSD); in particular, a retrospective study evidenced that depression was present in 30%–50% of military personnel with TBI or PTSD and anxiety episodes were reported in 60% of subjects within two years from the trauma [50]. Furthermore, considering them individually, in a study conducted by Chrisman et al., adolescents with a history of concussion had shown an increased risk 3.3 times greater of receiving a diagnosis of depression than those without previous medical history [51]. In the same way, states of anxiety are also linked to sleeping problems [51,52]. On the other hand, insomnia is associated with PTH as described by Jaramillo et al., observing that 27% of patients with PTH suffered from insomnia [52].

At present, the drug treatment of migraine offers a several kinds of remedies. Therapy should have efficacy and durability to restore the normal activities and do not present adverse events that are often associated with drug use [53].

### 4.1. NSAIDs (Non-Steroidal Anti-Inflammatory Drugs) Drugs

Non-steroidal anti-inflammatory drugs (NSAIDs) are the most represented drugs for migraine treatment and the first choice for medication [53]. In patients affected by PTH, where intracranial hemorrhage has been excluded, NSAIDs drugs such as ibuprofen or naproxen are often used for treatment. Petrelli et al. have conducted a study on 79 subjects (mean age 12.9 years) with PTH. The participants were divided into four groups on the pharmacological treatment: acetaminophen (*n* = 20), ibuprofen (*n* = 20), combined treatment with acetaminophen and ibuprofen (*n* = 19) and standard care (*n* = 20) without analgesic drugs. The combination treatment showed an efficacy of 79%, 61% for the ibuprofen group, 33% in the acetaminophen group and 21% in the standard care group. However, the use of NSAIDs drugs should be limited in order to reduce the possible risk of incurring in medication overuse (MO), while acetaminophen is used in subjects where the use of these drug treatment is not recommended [54].

### 4.2. Triptans Drugs

Triptans, a family of tryptamine-based drugs, represent a class of abortive medication for the acute therapy of migraine and cluster headache with proved efficacy [53]. The use of triptans, such as sumatriptan and rizatriptan, is advised in patients who do not respond to anti-inflammatory drugs and are characterized by migraine features [55] but are contraindicated in patients with vascular or coronary disease due to their vasoconstrictive effects [2]. A study conducted on military personnel with PTH, reported that the use of triptans medication induces a reliable headache 2 h post-doses if compared with military subjects that did not use triptans (*p* = 0.01). Although this is an observational clinic-based non-controlled study, these drugs could be effective for aborting acute attacks in subjects with PTH [56].

### 4.3. Tricyclic Antidepressants Drugs

Prophylaxis medications for migraine treatment are represented by Tricyclic antidepressant (TCAs) drugs [53]. The use of TCAs is recommended in patients presenting insomnia and PTH. Among the TCA drugs, amitriptyline is an effective antimigraine therapy, which induces sedation but presents adverse events such as dizziness, dry eyes and mouth, constipation and increased appetite and cardiac adverse events with onset arrhythmia and an extension of the QT interval [57]. The properties of amitriptyline include anti-anxiety and anti-depressant effects and sleep promoting which are characteristics of PTH [56]. Couch et al. described the efficacy of amitriptyline compared to the placebo group. In particular, the study showed a significant data regarding the treatment with amitriptyline at 8 weeks (*p* = 0.031) and at 16 weeks (*p* = 0.043). Furthermore, the amitriptyline group showed a significant decrease in headache frequency at 8 weeks if compared to the placebo (*p* = 0.018) [58]. A recent phase 2 clinical trial (NCT01856270) described the efficacy of amitryptyline in patients with mTBI. Participants, within the first 12 weeks after injury, were randomized into two groups: an immediate group (*n* = 24), which began administration drug directly after recruiting (one 10 mg capsule each evening), and an amitriptyline delayed group (*n* = 26), which began the pharmacological treatment on the day 30 visit (one 10 mg capsule each evening). The standard dosages were adjusted up to 25 mg during the second week and up to 50 mg during the third week of treatment. Only 33 subjects arrived at the end of 90 days of the therapy management. The results did not show a clarity preventive effect of amitriptyline for PTH disease [59].

### 4.4. Antiepileptics Drugs

Antiepileptic drugs are used as preventive treatment, including topiramate that can cause different side effects such as sleepiness, paresthesias, weight loss, metabolic acidosis, memory and/or language problems and renal dysfunction [60]. In a study conducted on military subjects with mild head trauma, topiramate showed a significant reduction in PTH frequency for patients, 57% suffering from chronic migraine and 31% presenting medication overuse. Results showed topiramate as an effective prophylactic treatment with respect to low doses of TCAs [56].

### 4.5. Monoclonal Antibodies

Treatment with anti-CGRP monoclonal antibodies inhibits hypersensitivity to migraine pain induced by glyceryl trinitrate (GTN), a known migraine factor [38]. They also proved to be effective in adults as a preventive treatment for migraine, showing an improvement in the disease from the first months of treatment [61]. A recent study described the efficacy of erenumab for the preventative treatment of migraine in patients with a clinical migraine phenotype following PTH. Subjects treated with erenumab showed a 95% reduction of headache days without serious adverse events. Further studies are needed to evaluate the efficacy and safety of erenumab in the treatment of PTH [62,63].

## 5. Discussion

Migraine is a primary headache disorder that afflicts millions of subjects, both adults and adolescents, while PPTH disorder is a common consequence of a TBI event affecting patients’ productivity and quality of life. Recently, many studies tried to understand the pathophysiological and metabolic mechanisms of PTH. ICHD-3 delineate the classification of PPTH after mTBI, and the clinical symptoms are not specific and have often been associated with anxiety and depression. FDA does not approve specific drug treatments for this condition and the development of adverse events or MO is very high. Different studies have focused on alterations in the brain structure, especially in the somatosensory region and the hypothalamus, identified through a magnetic resonance imaging analysis. The dynamic functional connectivity was significantly correlated with the frequency and intensity of headache in PPTH and migraine, respectively. Additionally, migraine disease is exacerbated by visual stimuli as photophobia while stress states intensify PPTH condition. The pathophysiological mechanisms underlying migraine and PPTH are not yet fully understood, and several data suggest that migraine and its chronic forms as drug-induced migraine showed altered levels of oxidative stress biomarkers following a reduction in antioxidant mechanisms. In addition to an increase in oxidative stress markers, mitochondrial metabolic alterations lead to high levels of lactic acid and a deficit of both NADH-dehydrogenase and cytochrome-c-oxidase. An increase in neural excitability at the cortex and trigeminal system levels could lead to an excessive use of migraine drugs and an increase in the perception of pain. There are really few data in literature regarding PTH acute or preventive pharmacological medications and, based on the results, the efficacy of the treatment could not be inferred. The first-choice medications for migraine are NSAIDs drugs but their use should be limited to avoid medication overuse (MO). Triptans drugs are contraindicated in patients with vascular or coronary disease due to their vasoconstriction effects while TCAs, used as prophylaxis medication, are indicated in patients with insomnia and PPTH. A few studies demonstrated that topiramate could be an effective prophylactic treatment compared to low doses of TCAs. In recent years, mAbs represented the new frontier of migraine treatment and the preliminary data shows an efficacy in the reduction of headache in subjects with PPTH treated with erenumab. The use of mAbs should be careful and carried out considering patients’ features like age, pubertal state and medical comorbidities.

## 6. Conclusions

PPTH is an important disorder with a high impact and, although it shows several comorbid symptoms, currently, there is no specific pharmacological therapy. The results presented in this review contribute to underline differences and similarities between migraine and PPTH. Further studies are needed to find targeted treatment and establish both safety and efficacy of the drugs. Pathophysiology of PTH and biomarkers implicated in the progression of PPTH should be better investigated. Further studies should consider pools of patients with as few interfering comorbidities as possible in order to attempt to randomize treatments, whether the latter are to be considered for acute or preventative management. This would allow a greater clarity on the therapeutic possibilities available for the therapist, and it would also allow to evaluate the possibility of different treatment lines both in the pathology free from comorbidity, free from contraindications and confounding factors, and in the case of comorbidity which may benefit from side effects of any medications in use. In this way, it would be possible to primarily treat PPTH and, in the same way, not interfere with any states of depression and/or anxiety, related or not to sleep disturbances.
